# EST-derived SSR markers used as anchor loci for the construction of a consensus linkage map in ryegrass (*Lolium *spp.)

**DOI:** 10.1186/1471-2229-10-177

**Published:** 2010-08-16

**Authors:** Bruno Studer, Roland Kölliker, Hilde Muylle, Torben Asp, Ursula Frei, Isabel Roldán-Ruiz, Philippe Barre, Céline Tomaszewski, Helena Meally, Susanne Barth, Leif Skøt, Ian P Armstead, Oene Dolstra, Thomas Lübberstedt

**Affiliations:** 1 Department of Genetics and Biotechnology, Faculty of Agricultural Sciences, Research Centre Flakkebjerg, Aarhus University, Forsøgsvej 1, 4200 Slagelse, Denmark; 2 Agroscope Reckenholz-Tänikon, Research Station ART, Reckenholzstr. 191, 8046 Zurich, Switzerland; 3 Institute for Agricultural and Fisheries Research (ILVO), Plant Sciences Unit - Growth and Development, Caritasstraat 21, 9090 Melle, Belgium; 4 Institut National de Recherche Agronomique (INRA) - UR4 Unité de recherche pluridisciplinaire prairies et plantes fourragères, BP6, 86600 Lusignan, France; 5 Crops Research Centre Oak Park, TEAGASC, Carlow, Ireland; 6 Institute of Biological, Environmental and Rural Sciences (IBERS), Aberystwyth University, Plas Gogerddan, Aberystwyth, Ceredigion SY23 3EB, UK; 7 Wageningen UR Plant Breeding, Wageningen University and Research Centre (PRI), P.O. Box 16, 6700 AA Wageningen, The Netherlands; 8 Department of Agronomy, Iowa State University, 1204 Agronomy Hall, 50011 Ames, IA, USA

## Abstract

**Background:**

Genetic markers and linkage mapping are basic prerequisites for marker-assisted selection and map-based cloning. In the case of the key grassland species *Lolium *spp., numerous mapping populations have been developed and characterised for various traits. Although some genetic linkage maps of these populations have been aligned with each other using publicly available DNA markers, the number of common markers among genetic maps is still low, limiting the ability to compare candidate gene and QTL locations across germplasm.

**Results:**

A set of 204 expressed sequence tag (EST)-derived simple sequence repeat (SSR) markers has been assigned to map positions using eight different ryegrass mapping populations. Marker properties of a subset of 64 EST-SSRs were assessed in six to eight individuals of each mapping population and revealed 83% of the markers to be polymorphic in at least one population and an average number of alleles of 4.88. EST-SSR markers polymorphic in multiple populations served as anchor markers and allowed the construction of the first comprehensive consensus map for ryegrass. The integrated map was complemented with 97 SSRs from previously published linkage maps and finally contained 284 EST-derived and genomic SSR markers. The total map length was 742 centiMorgan (cM), ranging for individual chromosomes from 70 cM of linkage group (LG) 6 to 171 cM of LG 2.

**Conclusions:**

The consensus linkage map for ryegrass based on eight mapping populations and constructed using a large set of publicly available *Lolium *EST-SSRs mapped for the first time together with previously mapped SSR markers will allow for consolidating existing mapping and QTL information in ryegrass. Map and markers presented here will prove to be an asset in the development for both molecular breeding of ryegrass as well as comparative genetics and genomics within grass species.

## Background

Ryegrasses (*Lolium *spp.) include the economically most important forage and amenity grass species and their economic value is likely to rise in future with increasing demand for meat and milk production and the development of environmentally friendly biofuels [1]. Perennial ryegrass (*Lolium perenne *L.) and Italian ryegrass (*Lolium multiflorum *Lam.) are naturally diploid (2n = 2x = 14) and outbreeding members of the Poaceaea family with a highly efficient two-locus self-incompatibility system. Current breeding methods are complemented by molecular genetic approaches, with genetic mapping as a prerequisite for marker-assisted selection and map-based cloning. In the case of perennial ryegrass, the International Lolium Genome Initiative (ILGI) reference mapping population [2] has been extensively characterised for a range of morphophysiological traits [3-7]. Additional mapping populations have been developed and characterised for various traits such as, VrnA (vernalization response, disease resistance, seed yield and fertility traits) [8-11], pop8490 (morphogenetic traits and resistance to crown rust) [12,13], WSC (water soluble carbohydrate accumulation and fertility traits) [7,14,15], TC1*SB2 (resistance to crown rust) [16], lpOA (resistance to crown rust and seed set), ZX (nitrogen use efficiency) [17] and F2 biomass (forage yield) [18]. The research focus for Italian ryegrass has been primarily on morphological and disease resistance traits [19-23].

Some of these mapping populations were evaluated for the same traits. Resistance to crown rust, for example, is one of the most important traits in ryegrass breeding. This is reflected in the number of populations in which this trait has been mapped (VrnA, pop8490, lpOA, Xtg-ART, TC1*SB2). Major and minor QTL for resistance to crown rust have been detected on all LGs in different mapping populations from both perennial [9,21,24-26] and Italian ryegrass [23], providing the opportunity to compare the source of resistance among mapping populations. Although some genetic linkage maps developed from these populations have been aligned with each other using publicly available markers [27], the number of common markers among genetic maps is very low, limiting the ability to infer cosegregation of QTL for a specific trait across populations. This is mainly due to the limited number of publicly available genetic markers for *Lolium *spp., and to some extent due to the limited transferability of markers across mapping populations. While traditionally a genetic map has been generated from a single population, recent efforts to create maps from multiple populations, referred to as consensus maps, have gained much interest in the scientific and breeding community. Integration of mapping data from individual maps into one consensus map has been reported in other forage [28] and cereal species [29-31] and aims at determining the relative positions of transferable markers in order to compare candidate gene and QTL locations across a broad variety of genetic backgrounds. A first effort towards a consensus linkage map in *Lolium *was based on two mapping populations [32] and used comparative RFLP probes as the core mapping set. Later, Jensen et al. [27] produced a consensus map from four mapping populations which contained 65 SSR markers. While this represented an improvement in terms of marker technology, some LGs were not adequately covered and large gaps were found on LG 5 and LG 6. Therefore, a large set of publicly available genetic markers with a high inter- and intraspecific amplification rate is crucial for map alignment, consensus map construction and, finally, for the assessment of co-location of QTL and candidate genes across populations.

SSR markers are hypervariable, multiallelic, often codominant, highly reproducible and, therefore, ideal to anchor molecular linkage maps [33]. Gene-associated SSRs derived from ESTs are of particular interest for linkage map alignments, since they are highly transferable to other pedigrees [34-36] and may functionally determine trait variation.

A large set of ryegrass EST-SSR markers has recently become available [37]. Here we report on the collective effort of seven European institutions (ART, Switzerland; DJF, Denmark; IBERS, United Kingdom; ILVO, Belgium; INRA, France; PRI, The Netherlands; and TEAGASC, Ireland) to i) provide the map positions of a large set of publicly available EST-SSRs, ii) to establish the first comprehensive consensus linkage map for *Lolium *spp. using EST-derived anchor SSR markers, iii) to complement this map with a reference set of publicly available SSR markers and iv) to assess the usefulness of EST-SSRs for comparative genetics across existing mapping populations in ryegrass.

## Results and discussion

### EST-SSR markers - a useful tool for comparative genetics and genomics

A total of 204 (43%) out of 464 recently published EST-SSR markers [37] have been assigned to map positions (additional file 1) using eight ryegrass mapping populations characterised for various traits (Table 1). Between 19 (LG 1) and 43 (LG 4) EST-SSRs mapped to each of the seven *Lolium *LGs and constitute a dedicated tool for comparative QTL mapping and map integration. EST sequences of 142 EST-SSRs (70% of the mapped EST-SSRs) revealed significant (E < e^-10^) sequence similarities in a BLASTX search against the non-redundant (nr) protein database of Genbank, out of which 89 (44%) correspond to genes with known functions (additional file 1). Protein functions were organised in seven groups representing genes with binding and catalytic activities (49% and 30%, respectively), transport activity (3%), enzyme regulatory activities (1%), as well as transcription and translation factors (7%) and structural genes (10%). These EST-SSRs are superior to random DNA markers for QTL mapping due to their putative functions [38,39]. Derived from more conserved transcribed genomic regions, EST-SSRs are more likely to be transferable to other mapping populations and grass species [34-36] and thus, are well suited as intra- and interspecific anchor loci and for cross-species phylogenetic studies [40].

**Table 1:** Detailed description of the mapping populations used for consensus linkage map construction and QTL analysis

Mapping population	Population design	Institution	Population size (number of individuals used for mapping)	Map reference	Number of mapped Gxx EST-SSRs	Traits assessed	Trait reference
ILGI	*Lolium perenne*, one-way pseudo-testcross (progeny of a cross between a di-haploid and a hybrid F1 plant)	IBERS, UK	183 (183)	[51]	3	Self incompatibility Plant fertility Plant morphology	[4] [7] [3]

VrnA	*Lolium perenne*, F2, two-way pseudo-testcross	DJF, DK	184 (172)	[8]	138	Vernalization response Crown rust resistance Powdery mildew Seed yield	[8] [9] [10] [11]

pop8490	*Lolium perenne*, F1, two-way pseudo-testcross	INRA, F	185(185)	[12,13]	40	Plant morphology Crown rust resistance	[12]

WSC	*Lolium perenne*, F2 (selfings of a single hybrid, obtained by crossing two partially inbred plants)	IBERS, UK	188 (188)	[32]	4	Water-soluble carbohydrates (sucrose, glucose and fructose) Plant fertility	[15] [7]

TC1*SB2	*Lolium perenne*, F1, two-way pseudo-testcross	ILVO, B	281 (281)	[16]	1	Crown rust resistance	[16] [25]

lpOA	*Lolium perenne*, F1, two-way pseudo-testcross	ILVO, B	147 (147)	Unpublished data	44	Crown rust resistance Seed set	Unpublished data

ZX	*Lolium perenne*, one-way pseudo-testcross (progeny of a cross between a di-haploid plant and LTS01)	PRI, NL	90 (90)	[17]	7	Nitrogen use efficiency	[17]

F2 biomass	*Lolium perenne*, F2 (selfings of a single hybrid, obtained by crossing two partially inbred plants)	TEAGASC, IRL	366 (363)	[43]	19	Segregation distortion Biomass	[43] [18]

Xtg-ART	*Lolium multiflorum*, F1, two-way pseudo-testcross	ART, CH	306 (96)	[19]	109	Bacterial wilt Crown rust resistance	[19] [23]

Mapping data of VrnA, Xtg-ART, lpOA, pop8490, F2 biomass, WSC and ILGI were combined for map integration and used for the construction of the ryegrass consensus map. The *Lolium multiflorum *Xtg-ART and the *Lolium perenne *TC1*SB2 were used to compare QTL for crown rust resistance.

A representative subset of 64 selected EST-SSR markers was further characterised and used to illustrate the relationships among the individual mapping populations. The number of identified alleles ranged from 2 to 14 with a mean value of 4.88. Between 21% (VrnA, WSC) and 69% (Xtg-ART) of the EST-SSRs were polymorphic in each mapping population. Overall, 83% of the markers were polymorphic in at least one mapping family.

### SSR consensus linkage map for ryegrass

**Figure 1:**
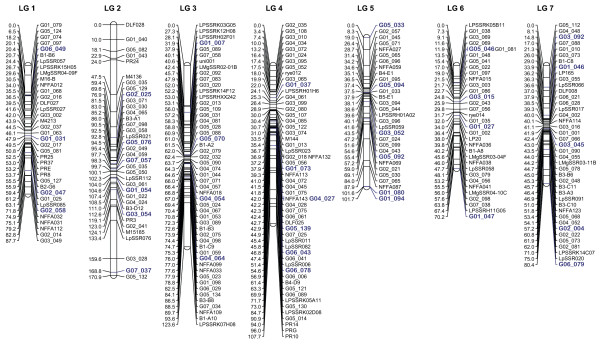
**A SSR consensus linkage map for ryegrass (*Lolium *spp.)**. The consensus linkage map was developed using the mapping populations VrnA, Xtg-ART, pop8490, lpOA, F2 biomass, WSC and ILGI (for detailed description of individual mapping populations, see Table 1). Mapping data were joined using the *Combine Groups for Map Integration *function of JoinMap 4.0 [58]. The Haldane mapping function based on regression mapping was used for map construction. The consensus linkage map contains 284 SSR markers. The total map length was 742 centiMorgan (cM), ranging for individual chromosomes from 70 cM of linkage group (LG) 6 to 171 cM of LG 2. The seven LGs have been aligned with the ILGI reference map [46]. Scale units are given in centiMorgan (cM) and *fixed order anchor loci *are highlighted in bold.

Integrated marker data of 204 EST-SSRs and 108 publicly available SSR markers were available for the construction of the consensus map. A total of 107 EST-SSRs were mapped in at least two mapping populations and served as anchor loci for map integration (15 anchor markers per LG on average, ranging from 7 on LG 1 to 23 on LG 4). Anchor markers with a highly conserved gene order across multiple populations, referred to as *fixed order anchor loci*, were used to define a *fixed order *for consensus mapping in JoinMap 4. On each LG, 4 to 6 *fixed order anchor loci *(a total of 35, highlighted in Figure 1), which were highly polymorphic, efficiently amplified by PCR and revealed easily detectable fragment sizes, were used to divide LGs into segments and provided the basis for the introduction of bins. These bins are representing defined chromosome regions of the *Lolium *genome, a highly useful concept already established in other major crop species [41,42].

However, the linear marker order between consensus and individual maps was not always congruent, particularly at closely linked marker loci. Inconsistencies in marker order were mainly due to differences in recombination frequencies of marker pairs in different populations. Such heterogeneous recombination frequencies occurred because the present study incorporated data collected from several mapping populations differing in design, size and marker density. For example, the proportion of distorted genetic markers differed between F1 and F2 designs. The percentage of markers showing distorted segregation (*P *< = 0.05) was highest for the F2 pseudo-testcross populations F2 biomass (10 out of 19, 53%) and VrnA (59 out of 138, 43%) and lowest for the F1 pseudo-testcrosses pop8490 and Xtg-ART with 3% (1 out of 38) and 8% (9 out of 109), respectively. Similar findings were recently reported by Anhalt et al. [43] who concluded that segregation distortion was most likely caused by genetic effects. Indeed, for the VrnA F2 population, the highest percentages of distorted markers were found on LG 1 (80%) and LG 2 (63%), clustering around the S and Z self-incompatibility loci located on those chromosomes [4]. This link between self-incompatibility and distorted F2 progenies has been shown earlier [11]. Interestingly, segregation distortion of the self incompatible species red clover (*Trifolium pratense *L.) was found to be specific for each accession anywhere in the genome.

For these reasons, map integration based on mean recombination frequencies and combined LOD scores using JoinMap 4.0 should be carefully interpreted [28,30,31] and the precise marker order may need to be verified in the population of interest. In order to ensure an accurate consensus marker order, *fixed order anchor loci *were used to define a *fixed order *for consensus mapping in JoinMap. Moreover, pairs of markers with a significant heterogeneity of recombination rates between populations were excluded. As a consequence, only 284 (91%) out of 312 available SSR loci were mapped in the final consensus map, i.e. 187 *L. perenne *EST-SSRs reported in Studer et al. [37], 21 EST-SSRs developed from *Festuca arundinacea *Schreb. [44], 18 genomic SSR markers derived from a *Lolium-Festuca *hybrid published by Lauvergeat et al. [45], 14 *L. perenne *genomic SSRs mapped by Jones et al [46], 16 *L. perenne *genomic SSRs published by Kubik et al. [47], 5 genomic SSRs derived from *L. multiflorum *[48] and 16, 3 and 4 genomic SSR markers of *L. perenne *developed at DJF, DvP and DLF-Trifolium, respectively, reported in Jensen et al. [27]. The consensus map covered a total genetic distance of 742 cM ranging from 70 cM of LG 6 to 171 cM of LG 2 (mean LG length of 106 cM) and contained 30 to 55 SSR markers (mean of 41) on each LG (Figure 1), a marker density useful for both comparative mapping and marker assisted breeding applications.

### Consistency of marker grouping and marker order

**Figure 2:**
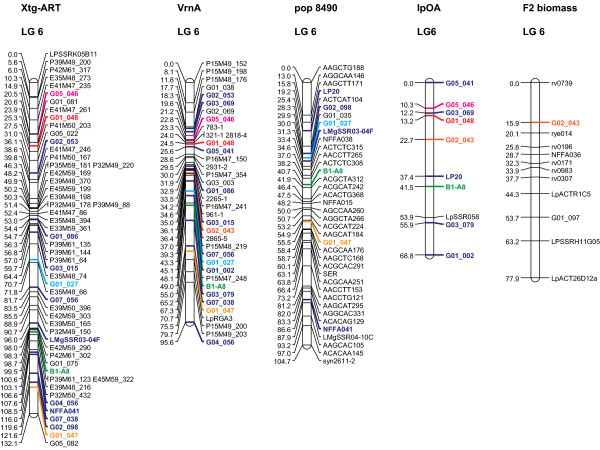
**Comparison of linkage group (LG) 6 between the mapping populations Xtg-ART, VrnA, pop8490, lpOA and F2 biomass**. The Haldane mapping function based on regression mapping of the software package JoinMap 4.0 [58] was used for map construction. Common markers used as anchors for map integration are indicated in blue bold, markers mapped in more than two populations share similar colours. Scale units are given in centiMorgan (cM).

**Figure 3:**
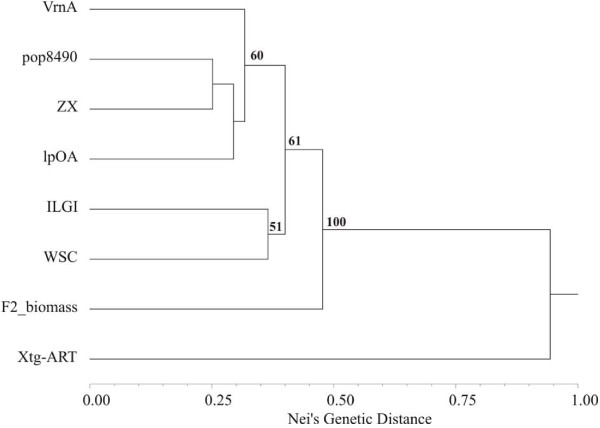
**UPGMA clustering of the mapping populations VrnA, pop8490, ZX, lpOA, F2 biomass, ILGI, WSC and Xtg-ART**. The dendrogram is based on Nei's genetic distance derived from allele frequencies of 64 EST-SSRs assessed in 6 to 8 individuals of each mapping population. Numbers above branches indicate percentage of bootstrap values obtained from 1000 re-sampling cycles.

The linear order of the markers in the individual maps was generally well conserved (as an example, see Figure 2). The VrnA map, which consisted of the highest number of mapped EST-SSRs and anchor markers, revealed a highly consistent marker order when compared to the consensus map. In contrast, the Xtg-ART map, with the second highest EST-SSR density, showed changed orders for some markers. Furthermore, the only inconsistencies in the assignment of EST-SSRs to LGs were observed in Xtg-ART, the Italian ryegrass population, while no inconsistency was observed between individual perennial ryegrass maps. G03_058 and G03_079 mapped on LG 2 and LG 6, respectively, in both VrnA and lpOA, but on LG 4 in Xtg-ART. Similarly, G01_075 mapped on LG 4 in VrnA and pop8490, but on LG 6 in Xtg-ART. G04_043 was assigned to LG 7 in Xtg-ART, but clearly grouped to LG 5 in VrnA and F2 biomass. Moreover, G03_028, G04_055 and G05_082 mapped to LG 4, LG 2 and LG 6 in Xtg-ART, respectively, but on LG 2 (G03_028 and G05_082) and LG 7 (G04_055) in VrnA. This might reflect chromosome rearrangements or - more general - differences in the genome organization between perennial and Italian ryegrass. Indeed, Xtg-ART was clearly separated from all perennial ryegrass populations in a UPGMA dendrogram based on Nei's genetic distance with a bootstrap value of 100% (Figure 3). Although some grouping of perennial ryegrass populations was observed, these groups were only supported by moderate bootstrap values. Still, the more closely related populations such as VrnA, lpOA and pop8490 were more consistent in terms of the marker order between maps.

### A dedicated tool for comparative QTL mapping

The small number of common markers between various genetic maps limits the ability to infer comparative positions of QTL across germplasm [7,9,49] and to associate interesting candidate genes to QTL detected in different mapping populations [50]. However, this is crucial not only to distinguish and address different sources of disease resistance in breeding, but also for the genetic characterisation of genomic locations conferring multiple pathogen resistance, as some QTL for disease resistance are commonly detected within similar chromosomal regions [49]. The current ryegrass consensus map provides the means to anchor maps across different pedigrees, to establish linkage with genes for agronomic traits and to compare QTL for important traits.

**Figure 4:**
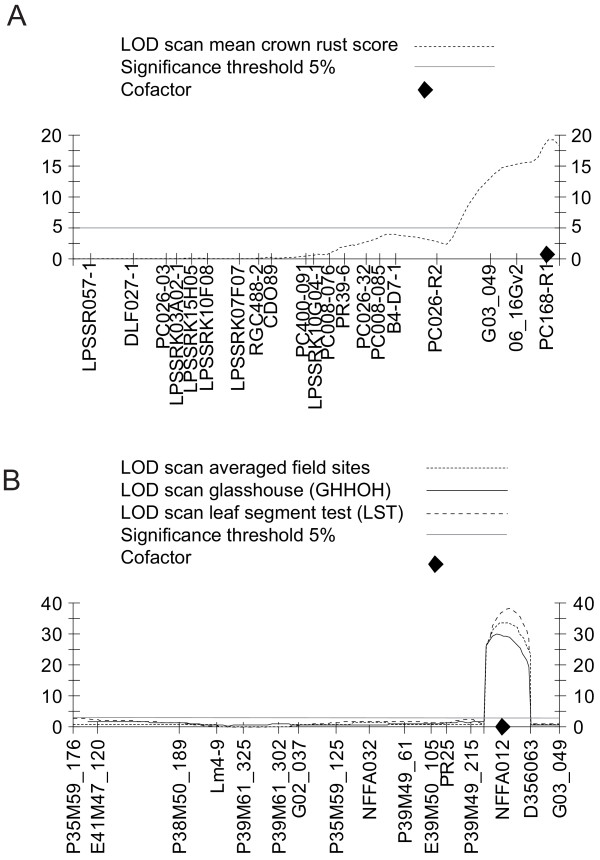
Genetic linkage map and LOD profiles of multiple QTL model mapping on linkage group (LG) 1 for resistance to crown rust assessed in the TC1*SB2 (A) and the Xtg-ART mapping population (B). For QTL analysis, the MapQTL 6 software [59] and 281 and 297 individuals of TC1*SB2 and Xtg-ART, respectively, were used to investigate crown rust related traits reported earlier [23,25]. The horizontal line indicates the LG-specific significance threshold. Cofactors are indicated and designate markers which absorb the genetic effects of their nearby QTL and function as a genetic background control.

In order to demonstrate the usefulness of anchored maps to compare QTL locations across mapping populations, the two major QTL for crown rust resistance on *Lolium *LG 1 detected in TC1*SB2 [25] and Xtg-ART [23] were used for comparative QTL mapping. Both located in the distal end of LG 1, the two QTL thus might represent the same source of resistance. EST-SSR G03_049 mapped at position 99 cM on LG 1 in the TC1*SB2 population, 10 cM away from the maximum LOD score value of the recalculated QTL explaining 30% of total phenotypic variation for resistance to crown rust at position 109 cM (Figure 4A). In contrast, QTL analysis in Xtg-ART detected NFFA012 at position 122 cM to explain the highest percentage of observed phenotypic variation for crown rust resistance [up to 38% for the trait "BLAST"; [23]], whereas G03_049 mapped at position 140 cM, clearly separated from the maximum of the QTL at position 135 cM (Figure 4B). Based on the QTL position relative to G03_049, the described QTL on LG 1 are likely to represent two distinct sources of crown rust resistance. The present consensus map indicates SSR markers that are located in this region and can be used for further mapping efforts. Moreover, the density of mapped EST sequences described in this paper also delivers a valuable resource for developing cross species genomic alignments, i.e., for cross-referencing between *Lolium*, and other grasses such as wheat, barley, rice and Brachypodium. These inter-species alignments are fundamental for transferring information between crop species and between crop and model species.

## Conclusions

This study has greatly increased the number of gene-derived SSR markers with known map positions as a tool for comparative QTL mapping in ryegrass and will facilitate a rapid transfer of linkage information between different ryegrass maps and eventually across related species. Overall, this consensus map, containing a large set of publicly available *Lolium *EST-SSRs, represents a major consolidation of existing ryegrass genetic mapping information and will prove to be an asset in the development of both molecular breeding for ryegrass and comparative genetics and genomics within the grasses.

## Methods

### Mapping populations

Eight ryegrass populations were used to assign EST-SSR markers to map positions: (i) the p150/112 intraspecific ILGI reference population consisting of 183 individuals [[51]; http://ukcrop.net/grass.html], (ii) 172 genotypes of the VrnA two-way pseudo-testcross population [8], (iii) 185 genotypes of the pop8490 two-way pseudo-testcross population [12,13], (iv) 188 F2 individuals of the WSC population [32], (v) 147 genotypes of the lpOA population, (vi) 90 genotypes of ZX population [17], (vii) 363 individuals of the F2 biomass population [43] and (viii) 96 individuals of the *L. multiflorum *Xtg-ART population [19]. These eight populations were selected based on their extensive use for genetic mapping and QTL analysis in ryegrass (Table 1).

### Genotyping of EST-SSR markers

Primer characteristics of EST-SSR markers along with the accession number and annotation of the corresponding *Lolium *EST and the PCR amplification protocols are described in Studer et al. [37]. The 143 EST-SSRs reported as being polymorphic in the VrnA mapping population were mapped using 172 VrnA F2 plants. Another set of 64 primer pairs was first evaluated for polymorphisms in six to eight individuals of each of the populations described above and then mapped in those populations for which clear polymorphisms were detected.

At each institute, PCR amplification and fragment separation were optimized for the technology available in-house. At DJF, the MegaBACE™ 1000 96 capillary electrophoresis system (GE Healthcare, Waukesha, WI) and the software GeneMarker version 1.6 (SoftGenetics, LLC., PA) was used to detect and score fragment sizes. At ART Reckenholz-Tänikon, amplification products were separated, visualised and scored using an ABI 3130 16 capillary electrophoresis system (Applied Biosystems, Foster City, CA) and the GeneMarker software version 1.5 (SoftGenetics, LLC., PA). The same capillary electrophoresis system was used at ILVO and TEAGASC, but in combination with the GeneMapper software version 4.0 (Applied Biosystems, Foster City, CA). At INRA and IBERS, M13-labelled tailed primers were used for PCR amplification [52] followed by electrophoresis on the LI-COR 4200 IR2 system (LI-COR, Lincoln, NE). The LI-COR system was also used at PRI, but with the adenine tail labelling method according to Marcel et al. [53].

### EST-SSR marker characterisation

The number of alleles was determined using the PowerMarker software [54]. Genetic divergence between the mapping populations was assessed using Nei's genetic distance [55] based on allele frequencies of 64 EST-SSRs assessed in 6 to 8 individuals of each population. A dendrogram was constructed using the UPGMA clustering method and bootstrap analysis with 1000 re-samplings implemented in NTSYSpc v. 2.2 [56]. The molecular function of mapped EST-SSRs was determined based on Gene Ontology (GO) using the Blast2GO search tool [57].

### Map construction

Map construction was carried out for each population separately using the independence LOD score for group formation and the Haldane mapping function based on regression mapping of the software package JoinMap 4.0 [58]. Individual LGs including all markers available from previous studies were calculated for each mapping population first. Markers with a mean chi-square contribution larger than five indicated that these loci did not fit very well at the respective map positions and were therefore excluded from further analyses. LGs from VrnA, Xtg-ART, lpOA, pop8490, F2 biomass, WSC and ILGI were subsequently joined using the *Combine Groups for Map Integration *function of JoinMap 4.0 [58]. Marker data of the individual populations were used to estimate all pairwise recombination frequencies and the corresponding LOD values. Combining the pairwise recombination values and LOD scores was possible by common markers that were shared by individual linkage maps. Such markers were considered as anchor loci, around which the map was developed. Differences in pairwise distance estimates of markers between populations were identified using the Heterogeneity Test of JoinMap 4.0 [58]. Since mapping was based on different population designs (i.e. F1 or F2) and the total number of mapped markers varied between populations, a consensus order of loci common between mapping populations was determined and used as *fixed order *for mapping. EST-SSR markers revealing such a conserved order between mapping populations were referred to as *fixed order anchor loci *and helped to overcome limitations of map integration based on averaged recombination frequencies and common LOD scores. The mapped EST-SSRs complemented with publicly available SSR markers were used for the final consensus map. The LGs are named according to the chromosome assignments in the ILGI reference population p150/112 which correspond to the homologous groups of the Triticeae cereals (Jones et al. 2002b).

### QTL analysis

Previously published QTL for resistance to crown rust in the mapping populations Xtg-ART [23] and TC1*SB2 [25] were recalculated including the EST-SSR G03_049, which was found to map in the vicinity of the QTL identified. QTL analysis was performed with MapQTL version 5.0 [59] using multiple QTL mapping (MQM). Automatic cofactor selection (backward elimination, *P *< 0.02) was used for the detection of significantly associated markers as cofactors. LOD significance threshold levels were determined using 1,000 permutations.

## Authors' contributions

TL, TA and UF conceived the study. BS coordinated the study, participated in its design, collected the mapping data, performed the linkage mapping, drafted and coordinated the work on the manuscript. BS, RK, HMU, PB, CT, HM, LS, OD, SB and IPA provided the mapping data of the mapping populations. RK and BS carried out the statistical analysis. RK, HMU and BS recalculated QTL for crown rust resistance in the mapping populations Xtg-ART and the TC1*SB2. All authors read and approved the final manuscript.

## Supplementary Material

Additional file 1 **Detailed mapping information of EST-SSR markers**. This table originally published by Studer et al. [37], was supplemented with detailed mapping information such as the linkage group where the markers map to, the map position in each mapping population and the information, if a marker was used as an *anchor *or a *fixed order anchor locus*. Click here for file
